# Ebola Virus Disease Survivors Show More Efficient Antibody Immunity than Vaccinees Despite Similar Levels of Circulating Immunoglobulins

**DOI:** 10.3390/v12090915

**Published:** 2020-08-20

**Authors:** Till Koch, Monika Rottstegge, Paula Ruibal, Sergio Gomez-Medina, Emily V. Nelson, Beatriz Escudero-Pérez, Matthias Pillny, My Linh Ly, Fara Raymond Koundouno, Joseph Akoi Bore, N’Faly Magassouba, Christine Dahlke, Stephan Günther, Miles W. Carroll, Marylyn M. Addo, César Muñoz-Fontela

**Affiliations:** 1 Division of Infectious Diseases, 1st Department of Medicine, University Medical Center Hamburg-Eppendorf, 20251 Hamburg, Germany; t.koch@uke.de (T.K.); ly@bnitm.de (M.L.L.); dahlke@bnitm.de (C.D.); m.addo@uke.de (M.M.A.); 2 Bernhard Nocht Institute for Tropical Medicine, 20359 Hamburg, Germany; rottstegge@bnitm.de (M.R.); paularuibal@gmail.com (P.R.); gomez-medina@bnitm.de (S.G.-M.); nelson@bnitm.de (E.V.N.); beatriz.escudero@bnitm.de (B.E.-P.); guenther@bni.uni-hamburg.de (S.G.); 3 German Center for Infection Research, partner site Hamburg-Lübeck-Borstel-Riems, 20359 Hamburg, Germany; 4 Clinical Psychology and Psychotherapy, Institute of Psychology, Faculty of Psychology and Human Movement Science, University of Hamburg, 20146 Hamburg, Germany; matthias.pillny@uni-hamburg.de; 5 Ministry of Health Guinea, 2101 Conakry, Guinea; koundounofr@yahoo.fr (F.R.K.); jabore34@gmail.com (J.A.B.); 6 Université Gamal Abdel Nasser de Conakry, 2101 Conakry, Guinea; cmagassouba01@gmail.com; 7 Research and Development Institute, National Infection Service, Public Health England, Porton Down, Salisbury, Wilts SP40JG, UK; Miles.Carroll@phe.gov.uk

**Keywords:** Ebola virus, VSV, vaccine, antibodies, immune memory

## Abstract

The last seven years have seen the greatest surge of Ebola virus disease (EVD) cases in equatorial Africa, including the 2013–2016 epidemic in West Africa and the recent epidemics in the Democratic Republic of Congo (DRC). The vaccine clinical trials that took place in West Africa and the DRC, as well as follow-up studies in collaboration with EVD survivor communities, have for the first time allowed researchers to compare immune memory induced by natural infection and vaccination. These comparisons may be relevant to evaluate the putative effectiveness of vaccines and candidate medical countermeasures such as convalescent plasma transfer. In this study, we compared the long-term functionality of anti-EBOV glycoprotein (GP) antibodies from EVD survivors with that from volunteers who received the recombinant vesicular stomatitis virus vectored vaccine (rVSV-ZEBOV) during the Phase I clinical trial in Hamburg. Our study highlights important differences between EBOV vaccination and natural infection and provides a framework for comparison with other vaccine candidates.

## 1. Introduction

Ebola virus disease (EVD) is a severe hemorrhagic fever caused by viruses of the genus *Ebolavirus*, more prominently by Ebola virus (species *Zaire ebolavirus*, EBOV). EBOV was the causative agent of the 2013–2016 EVD epidemic in West Africa as well as the recent epidemic in the Democratic Republic of Congo (DRC) [1]. The magnitude of the EVD epidemic in West Africa allowed for the first clinical studies, the establishment of clinical trials to test vaccines and therapeutics as well as investigations on post-Ebola syndrome. These research efforts have significantly improved our understanding of EVD pathophysiology and immunity. Regarding the latter, findings from several laboratories indicate that acute EVD is characterized by exacerbated and sustained adaptive immune responses [2,3,4] and that both arms of the adaptive immunity are critical for recovery [5,6,7].

Antibodies exert several complementary functions during the immune response to pathogen invasion. These functions rely on the structure of the antibody molecule, consisting of the variable region (Fab) which harbors a hypervariable antigen-binding site as well as a constant region (Fc). The latter can engage the complement system and Fc receptors (FcγR) in many immune cells, including natural killer (NK) cells as well as a variety of phagocytes. Thus, antibodies can trigger a wide range of immune responses to viruses including neutralization of the ability of the virus to enter or exit target cells, activation of infected cell phagocytosis, antibody-dependent cellular cytotoxicity (ADCC) and complement-mediated cytotoxicity (CMC) [8,9].

Since the characterization of the first monoclonal antibody against EBOV, KZ52 isolated from a survivor of the Kikwit outbreak [10], there has been substantial research devoted to the study of the functional properties of anti-EBOV antibodies isolated from EVD survivors. Perhaps surprisingly, these studies demonstrated that even though neutralizing antibodies (N_AB_) are maintained for years after recovery [11,12], neutralization of EBOV in cell culture may or may not translate into protection [13,14]. The maintenance of serum anti-ebolavirus antibodies with the capacity to bind to FcγR for up to a decade suggests that, in addition to neutralization, FcγR-dependent functions are also an important component of immune memory [12]. Indeed, monoclonal antibody therapy, which has shown promise as a successful post-exposure therapy, is based on the administration of antibody cocktails that include neutralizing and non-neutralizing antibodies. A recently developed human antibody cocktail with Fab- and FcγR-dependent biological functions has shown promising pan-*Ebolavirus* protection [15]. In agreement with the idea that multiple antibody functions are necessary to survive EVD, N_AB_ are often not detected in survivors during the early weeks after discharge from the treatment center [16,17].

The magnitude of the 2013–2016 West African EVD outbreak led to the launch of clinical trials for EBOV vaccines that had shown strong efficacy in non-human primate models. Most notably, the recombinant vesicular stomatitis virus (rVSV)-vectored vaccine (rVSV-ZEBOV), in which the VSV envelope glycoprotein (GP) is replaced by EBOV GP, showed evidence of protection in ring vaccination-based clinical trials conducted in West Africa and the DRC [18], and it is now licensed for human use in the US [19]. Despite its success, our understanding of the host immunity that builds after rVSV-ZEBOV vaccination is incomplete, and, to the best of our knowledge, the study of the biological properties of the antibodies generated by rVSV-ZEBOV vaccination has been limited to neutralization [20,21,22]. However, other vaccine candidates have been shown to induce Fc-mediated antibody functions such as NK cell activation [23].

The purpose of this study was to explore immune memory induced by natural infection and vaccination against EVD. We compared the antibody functions of 10 rVSV-ZEBOV vaccinees and 25 EVD survivors 6 months after vaccination or approximately 12 months after discharge, respectively, for differences in anti-EBOV glycoprotein antibodies. An improved understanding of immune responses in disease and vaccination might ultimately aid in the development of novel therapeutics and vaccines.

## 2. Materials and Methods

### 2.1. Sample Preparation

Plasma samples were collected 180 days after a single vaccination with 2 × 10^7^ plaque-forming units (PFUs) of rVSV-ZEBOV as described in the study protocol EudraCT No. 2014-003591-23, ClinicalTrials.gov No. NCT02287480 [24]. The mean age of vaccinees was 40 years of age. Plasma samples of long-term (~1 year) EVD survivors were collected in Guéckédou, Guinea, after receiving written consent. Samples were collected under Ethics protocols approved by the Guinean National Committee for Research and Health (33/CNERS/15) and the Medical Ethics Commission of the state of Hamburg (PV5309). For the survivor cohort, mean age was 32 years (see Table 1). Plasma samples were aliquoted and stored at −80 °C. All samples from Guéckédou were cooled and transported to Donka hospital in Conakry where they were stored at −80 °C. After that, survivor plasma samples were transported to Hamburg in dry ice.

### 2.2. Plaque Reduction Neutralization Assay (PRNA)

PRNA was performed using donor plasma dilutions incubated with 100 focus-forming units (FFUs) of EBOV H.sapiens-tc/COD/1976/Yambuku-Mayinga strain for 1 h at room temperature (RT). These dilutions were then plaqued on 90 to 100% confluent Vero-E6 cell monolayers. Cells were overlaid with minimum essential medium (MEM) containing 0.64% agarose (Oxoid) and the corresponding plasma dilutions. After 4 days of incubation at 37 °C, the cells were fixed with 3.7% paraformaldehyde (PFA), and the plaques were stained using a polyclonal anti-EBOV mouse serum (1:1000), an anti-mouse secondary antibody conjugated to horseradish peroxidase (HRP) (Sigma-Aldrich, St. Louis, MO, USA) and Trueblue reagent (Sera Care, Milford, MA, USA). Plaques were counted in each plasma dilution, and the percent inhibition for each plasma sample and dilution was individually calculated based on a no-antibody control. All experiments with infectious EBOV were carried out in the biosafety level 4 laboratory of the Bernhard Nocht Institute for Tropical Medicine in Hamburg, Germany, according to institutional biosafety regulations. All personnel handling samples wore positive-pressure biosafety suits.

### 2.3. rVSV ZEBOV Vaccine and Recombinant Proteins

The vaccine was manufactured at IDT Biologika in Dessau, Germany, and stored in a manner consistent with good manufacturing practices. The stock of 1 × 10^8^ plaque forming units (PFUs)/mL was further diluted as required. Recombinant Ebola virus glycoprotein (GP) minus the transmembrane region (EBOV rGPdTM) was purchased from IBT Bioservices (Rockville, MD, USA).

### 2.4. Generation of Antigen-Coupled Beads and Isotyping Assay

Magnetic carboxylated fluorescently labeled microbeads (Magplex beads, Luminex Corp., Austin, TX, USA) were coupled with Ebola-GP (EBOV rGPdTM by IBT Bioservices). The coupled beads were utilized in a multivariate Luminex assay for antibody class determination. Briefly, beads were diluted to a concentration of 50 beads/μL in Assay Buffer (PBS-1×, 0.1% BSA). Using a black, clear bottom 96-well plate (Greiner Bio One, Kremsmünster, Austria Cat. No. 655906), 50 μL of the working microsphere mixture (2500 beads/well), 40 μL of Assay Buffer and 10 μL of plasma sample diluted 1:10 (1 µL of plasma) were added to each well. The plate was covered and incubated overnight at 4 °C. A Bio-plex array reader (Bio-Plex 200, Bio-Plex Manager 5.0, Bio-Rad Laboratories, Inc., Hercules, CA, USA) detected the microspheres, and binding of PE detector antibody was measured to calculate a Median Fluorescence Intensity (MFI). Background signal, defined as the average MFI observed for each microsphere set when incubated with the PE detector antibody in the absence of a clinical antibody sample, was subtracted from the MFI for each sample. The human anti-EBOV-GP antibody KZ52 (IBT Bioservices Cat. No. 0260-001) was used as a positive control.

### 2.5. ADCC and Antibody-Dependent Cellular Phagocytosis (ADCP) Reporter Assays

ADCC was measured using the ADCC Reporter Bioassay kit (Promega, Madison, WI, USA) according to the manufacturer’s instructions. ADCP was measured using the FcyRIIa-H Reporter Kit (Promega). Luminescence was measured immediately using a Synergy Hybrid Reader (BioTek Instruments, Inc., Winooski, VT, USA).

### 2.6. NK Degranulation Assay

NK cell activation was measured by the ability of EBOV-GP specific antibodies to induce expression of CD107a on human NK cells. High-binding bottom ELISA plates (Greiner Bio One, Cat. No. 705073) were coated overnight with 300 ng/well EBOV-GP at 4 °C. Wells were washed twice with PBS, blocked overnight with PBS containing 5% fetal calf serum at 4 °C and washed twice again. Wells were then incubated with 100 µL of a 48-fold dilution of patient’s plasma for 2 h at 37 °C and washed again. Cryopreserved human PBMCs from one healthy individual were thawed, washed and picked up in RPMI 1640 medium containing low IgG Serum. A total of 3 × 10^5^ PBMCs were added to each well and incubated overnight at 37 °C. Cells were then stained for CD3-AF700, CD45-Pacific Blue and CD107a-PE for 1 h at 4 °C, washed, picked up in 1% PFA and measured on a LSR-II flow cytometer (BD Biosciences, Franklin Lakes, NJ, USA). Degranulated NK cells were defined as CD107a^+^ CD3^−^ CD56^+^ cells. Analysis was performed using FlowJo X 10 software (FlowJo LLC, Ashland, OR, USA).

### 2.7. Statistical Analysis

Statistical analysis was performed with Prism GraphPad version 7.03 (GraphPad Software, San Diego, CA, USA). Non-parametric statistics were applied throughout. For group comparisons we utilized Kruskal–Wallis analysis followed by Dunn’s post-test. To evaluate putative correlations, we utilized Spearman’s correlation analysis.

## 3. Results

### 3.1. EVD Survivors Show Enhanced Antibody Functions in Comparison with Vaccinees

**Figure 1:**
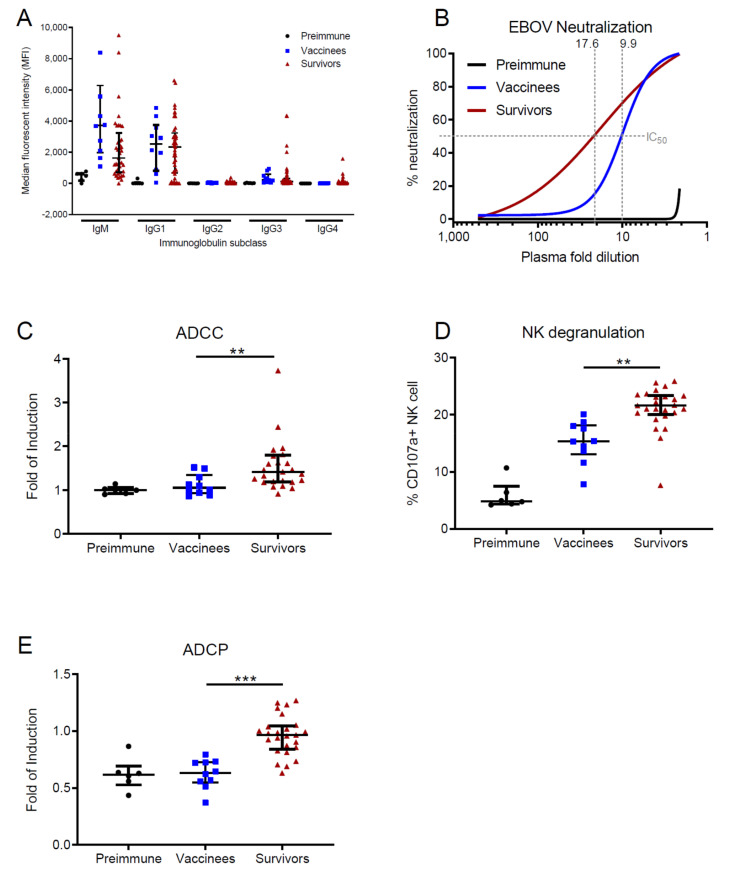
Antibody quantity and functions in recombinant vesicular stomatitis virus vectored vaccine (rVSV-ZEBOV) vaccinees and Ebola virus disease (EVD) survivors. Plasma samples of individuals vaccinated with 2 × 10^7^ PFUs of candidate vaccine rVSV ZEBOV 180 days after vaccination (vaccinees, *n* = 10, blue squares) were compared with plasma from EVD survivors (survivors, *n* = 25, red triangles). Median Ig levels of vaccinees before vaccination (preimmune, *n* = 6, black points) served as a control. Points show the individual measurements. Bars show the median as well as first and third quartile of the sample distribution. (**A**) Beads based ELISA for analysis of immunoglobulin (Ig) isotypes and subclasses. Shown is the Median Fluorescent Intensity (MFI) of the secondary antibody in logarithmic scale. (**B**) Plaque reduction (neutralization) assay using authentic EBOV (Mayinga variant). Lines indicate the nonlinear regression. IC_50_ values are indicated by light grey pointed lines. (**C**) Antibody-dependent cellular cytotoxicity (ADCC) reporter assay. (**D**) Natural killer (NK) cell degranulation assay. (**E**) Antibody-dependent cellular phagocytosis (ADCP) reporter assay. In all graphs, significance is indicated in lines. Significance levels are calculated using Mann–Whitney tests and indicated by asterisks, namely ** *p* ≤ 0.01; *** *p* ≤ 0.001. IC_50_: half maximal inhibitory concentration.

To characterize the presence of anti-EBOV antibodies in survivors and vaccine recipients in the long-term after recovery and vaccination, we first sought to determine the overall levels of EBOV-GP specific IgM and IgG isotypes as well as the four IgG subclasses, since the distribution of the latter may provide cues on the breadth of antibody functions in immune memory [25]. To this end, we set up a microsphere (“beads”)-based array allowing for on-bead affinity purification and subsequent classification of antigen-specific antibodies from clinical samples (see assay description in Figure S1). Surprisingly, both survivors and vaccinees showed significant levels of serum IgM 1 year and 180 days post-vaccination, respectively (vaccinees: median = 3712, Q_1_ = 1977, Q_3_ = 6279 Median Fluorescence Intensity (MFI); survivors: median = 1623, Q_1_ = 724, Q_3_ = 3241 MFI; Figure 1A). As expected, the most abundant IgG subclass in serum was IgG1 (median of IgG1, IgG2, IgG3 and IgG4 in vaccinees: 2521, 35, 241 and 16 MFI; survivors: 2339, 43, 122 and 21 MFI, respectively). Overall, there were no significant differences between survivors and vaccinees regarding the levels of circulating Ig subclasses, and the levels resembled those seen in survivors from other studies [6].

To analyze the quality of the humoral immune response in survivors and vaccinees, we performed four different quality assays. To test Fab-mediated Ab functions, we determined the N_AB_ titers in survivor and vaccine recipient plasma samples using a plaque reduction neutralization assay (PRNA) with authentic EBOV. The plasma samples from survivors showed a higher neutralization capacity than that of vaccinees (Figure 1B and Figure S2) (median IC_50_ survivors: 17.6 (95%CI 0 to 37.03); median IC_50_ vaccinees: 9.9 (95%CI 7.979 to 12.42) fold dilution, respectively), which suggests that even when compared with high-dose vaccination, surviving EVD generates a broader pool of N_AB_. Next, we sought to determine the quality of FcγR-dependent functions in both cohorts. To this end, we performed two assays to quantify ADCC and one additional assay to determine antibody-dependent activation phagocytosis (ADCP). Both ADCC assays (see Methods Section) revealed a higher capacity to induce NK cell activation in survivors than in vaccinees (Figure 1C,D). Similarly, survivor plasma samples were more efficient at inducing ADCP (Figure 1E).

Taken together, these data indicate that natural infection leads to a more efficient antibody immunity than vaccination, which is reflected in a higher capacity of virus neutralization and activation of cellular responses.

### 3.2. Antibody Functions Are Associated with Viral Loads in Survivors

**Figure 2:**
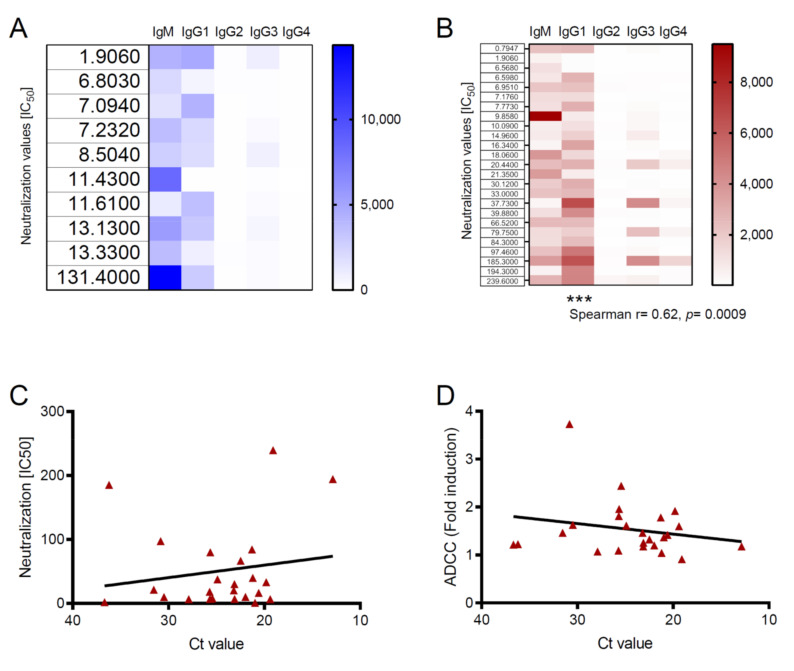
Correlation analysis. Spearman correlation analysis between the plaque reduction neutralization assay (PRNA) values and Ig subclass in vaccine recipients (**A**) and EVD survivors (**B**). Significance is depicted as *** *p* ≤ 0.001. (**C**,**D**) Spearman correlation analysis between PCR C*t* values and neutralization values (**C**) or ADCC fold induction (**D**) in individual EVD survivors. Lines represent linear regression.

An interesting point in our study was the finding that, despite similar levels of circulating Ig, antibody functions were enhanced in survivors compared to vaccinees. These results suggested that specific Ig subclasses triggered more efficient immune responses in survivors than vaccine recipients. To investigate this, we performed multiple correlation analyses where we searched for associations between Ig subclasses and Ab functions. As shown in the heatmaps of Figure 2A,B, the levels of IgG1 correlated positively with virus neutralization in survivors but not in vaccinees. Indeed, neutralization was the only antibody function that showed a positive correlation with an Ig subclass; ADCC and ADCP did not (Figure S3 available online).

Previous studies into other viral infections have found positive correlations between virus loads during the acute phase of infection and the subsequent neutralizing antibody activity [26,27]. Since natural EBOV infection presents different degrees of severity that are correlated with the viral loads [28,29], we next sought to assess the relationship between pathogen loads and virus neutralization in EVD. Linear regression analysis showed a positive association between virus loads (represented as PCR C*t* values) and EBOV neutralization titers. However, this association did not reach statistical significance in the correlation analysis (Figure 2C). Interestingly, we observed the opposite trend when we evaluated the relationship between virus loads and ADCC. Although further research is needed to draw conclusions in this regard, this finding could indicate that Fc-dependent functions are more effective in survivors that showed low levels of viremia during the acute phase of EVD (Figure 2C).

## 4. Discussion

In this study we compared the levels of EBOV-specific antibodies and the antibody-mediated memory immune responses in EVD survivors and rVSV-ZEBOV vaccinees long-term after discharge (one year) and vaccination (6 months), respectively. Both survivors and vaccinees sustained similar levels of serum anti-EBOV Ig, but the antibody-mediated immune responses were superior in survivors. As shown for other infectious diseases [30,31], the levels of circulating antibodies do not necessarily correlate with the strength of the humoral response against EBOV. In fact, the correlates of protection (CoP) against EVD have not been fully established [32]. One reason for this is that immunity after EVD or vaccination can only be examined in an outbreak scenario. Another reason is that immunity after vaccination in animal models, such as rodents or nonhuman primates (NHPs), does not always correlate with findings in humans. As an example, while anti-EBOV IgG titers correlated with protection in rodents and NHPs [33], in humans the IgG level might be more of a surrogate parameter (i.e., non-mechanistic CoP) for the mechanistic CoP of neutralizing IgM antibodies [20].

While antibodies are considered an integral part of the immunity built against EBOV after disease and vaccination [34], cellular immunity, particularly CD8+ T cells, is also considered integral for protection [32]. Lastly, innate immune responses such as NK cell activity might offer another surrogate CoP [35].

Recently, some authors have suggested that different CoP might have to be utilized for different EBOV vaccines [36] depending, among other parameters, on the antigen used and the route of vaccination. Thus, we need to apply caution when using solely antibody titration to assess the effectiveness of putative vaccines, as this alone may not reflect immunity.

Our study shows that, in both survivors and vaccinees, EBOV-specific IgM is maintained long after exposure, which is consistent with previous findings in rVSV-ZEBOV vaccine recipients [20] and survivors [6]. In this previous rVSV-ZEBOV vaccine study, IgM was predominant even after the second vaccination and played an important role in virus neutralization. Long-lived IgM levels produced by germinal center-independent plasma cells have been previously described and demonstrated a contribution to protection against pathogen challenge [37,38,39]. In future studies it would be interesting to dissect the specific role of these IgM-producing plasma cells in EBOV immunity since, in some instances, they may be maintained for a lifetime [37].

The acute phase of EVD is associated with high levels of circulating virus, systemic dissemination of the pathogen and high levels of inflammation, which may result in dysregulation of cellular immunity [3], endothelial dysfunction [40] and secondary sepsis [41], among other complications. Some previous studies into animal models have indicated that EBOV infection causes severe lymphocytolysis in germinal centers which are necessary for B cell maturation [42]. Yet all the antibody functions were more effective in survivors than in vaccinees that received a controlled, vector-delivered dose of EBOV GP. These findings indicate that, despite all the immune disturbances associated with acute EVD, survivors were able to mount effective B cell-mediated immunity. Since most individuals are immunologically naïve prior to EBOV infection, these results also highlight the importance of making a successful transition between innate and adaptive immunity to survive EVD. To better understand how adaptive immune responses are initiated in response to infection and vaccination, it would be interesting for future studies to compare how antigen-presenting cells process antigens carried by VSV and authentic EBOV.

Previous studies have identified a positive correlation between virus loads and the strength of antibody-mediated neutralization [26,27], although this does not necessarily apply for all viral infections [43]. Antigen availability and the quality of CD4 T cell-mediated help are critical to induce B cell maturation [44], which suggests that survivors were able to maintain CD4 T cell immunity and successful rounds of B cell affinity maturation in the presence of high virus loads. Even though we do not have data on B cell clonality, we also speculate that, even in the presence of high loads of EBOV, the B cell response may be dominated by a few epitopes. This is because the availability of many epitopes reduces the affinity and diversity of the antibody repertoire [45].

The role of ADCC and other Fc-mediated antibody functions in EVD is poorly understood. It is becoming clear however that, in addition to neutralization, ADCC may significantly contribute to EBOV clearance [46,47]. The finding that the strength of ADCC is associated with low viremia is notable and may have different explanations. For example, ADCC may contribute significantly to control viremia during the acute phase of EVD; therefore, survivors show antibodies that retain ADCC function during the memory response. In addition, it is conceivable that the levels of antigen and the associated inflammation may polarize the antibody response towards Fab- or Fc-dependent functions. Finally, the breadth of the antibody repertoire and the epitope coverage may significantly influence antibody functions. In this regard, anti-EBOV GP antibodies exhibit a different capacity of FcγR function engagement which depends on the epitope to which they bind. Anti-GP mAbs targeting membrane proximal epitopes or the GP mucin domain do not rely on Fc–FcγR interactions to confer activity, whereas antibodies against the GP chalice bowl and the fusion loop require FcγR engagement for optimal in vivo antiviral activity [48]. Further experiments in adequate animal models would be very valuable in determining how acute EBOV infection shapes subsequent antibody responses.

The limitations of this study are the small sample sizes, especially for the vaccinee cohort, and differences in age, sex and ethnicity as reported in Table 1. The differences in timepoints between the two cohorts that were available for analysis (6 months post vaccination for vaccinees, approximately one year after recovery for EVD survivors) might also provide a confounding factor.

Our study highlights important immunological differences between vaccination and natural EBOV infection and provides evidence that antibody titers alone may not be sufficient to evaluate vaccination-induced antibody immunity. Comparisons between immune memory in survivors and vaccinees provide a unique opportunity to compare the immunogenicity of current vaccine candidates as well as future vaccines.

## Figures and Tables

**Table 1:** Study cohort. Age, sex and ethnicity of rVSV-ZEBOV vaccinees at d0 and of EVD survivors on day of blood sampling are shown. Absolute numbers (%) are displayed unless otherwise specified.

	rVSV-ZEBOV Vaccinees (*n* = 10)	EVD Survivors (*n* = 25)
**Age, mean (SD) (y)**	40.4 (8.7)	32.0 (12.9)
median (range) (y)	40 (24–52)	31 (18–58)
**Sex**		
female	3 (30%)	11 (44%)
male	7 (70%)	14 (56%)
**Ethnicity**		
white	10 (100%)	0
Black or African American	0	10 (100%)

## Supplementary Materials

The following are available online at https://www.mdpi.com/1999-4915/12/9/915/s1, Figure S1: Principle and quality control of the subclassing assay. Figure S2: Donor-specific EBOV neutralization curves. Figure S3: Analysis of correlation between Ig subclasses, ADCC and ADCP.
