# Supplementary material for: Ebola Virus Disease Survivors Show More Efficient Antibody Immunity than Vaccinees Despite Similar Levels of Circulating Immunoglobulins
Source: Viruses. 2020 Aug 20;12(9):915. doi: 10.3390/v12090915 (PMC7552031; doi:10.3390/v12090915)
Supplement: Supplementary file 1 [file viruses-12-00915-s001.zip › viruses-867717-supplementary.pdf]

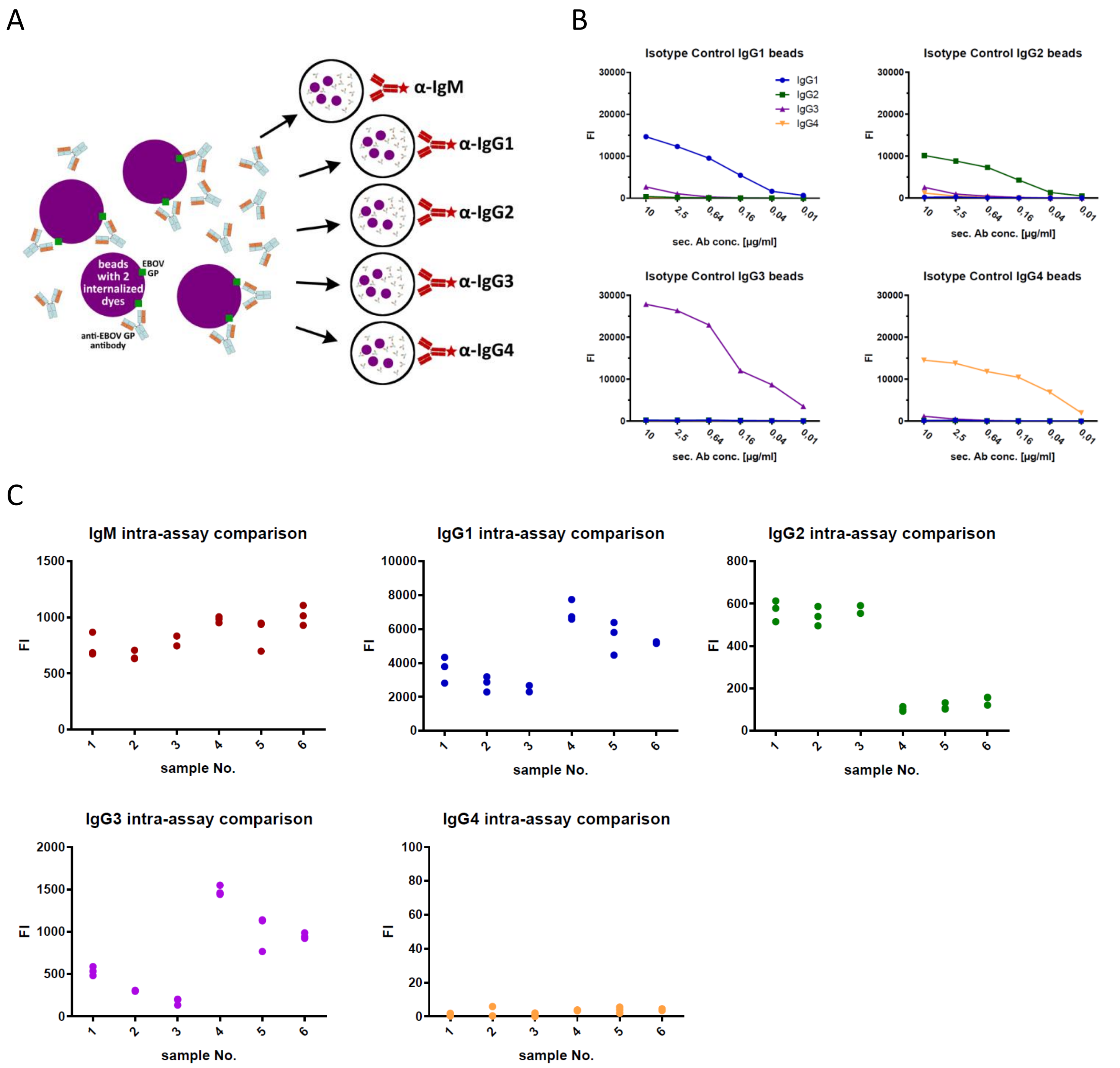

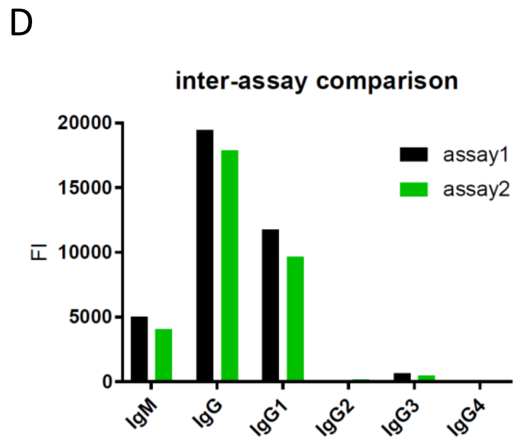

Figure S1: Principle and quality control of the subclassing assay. (**A**) Principle of the Assay Beads based ELISA for analysis of Immunoglobulin (Ig) isotypes and subclasses. Magnetic fluorescent microbeads are labeled with Ebola-GP, incubated with sample plasma, split into replicate wells and stained with secondary anti-human Immunoglobulin (Ig) Phycoerythrin- (PE-) labelled antibodies that are specific for a certain Ig subclass. (**B**) Control of the secondary antibodies: beads were coupled with Isotype controls of the four human IgG subclasses and incubated with the different secondary antibodies (sec.Ab). At the concentration the sec. Ab were used [0,64µg/ml], no cross-reactivity between the four IgG subclasses was observed. (**C**) intra-assay control: triplicates of sample wells were analyzed after incubation with each of the five sec. Ab. (**D**) inter-assay control: Assay1 was repeated with the same samples in triplicates after one month (assay2).

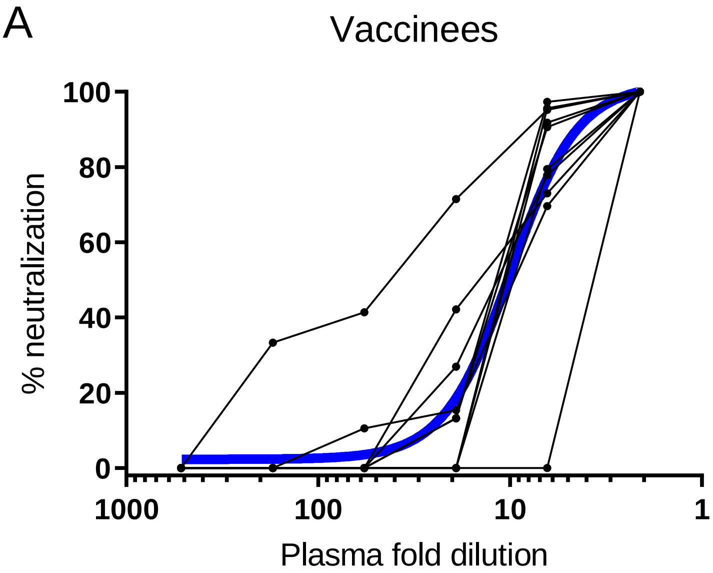

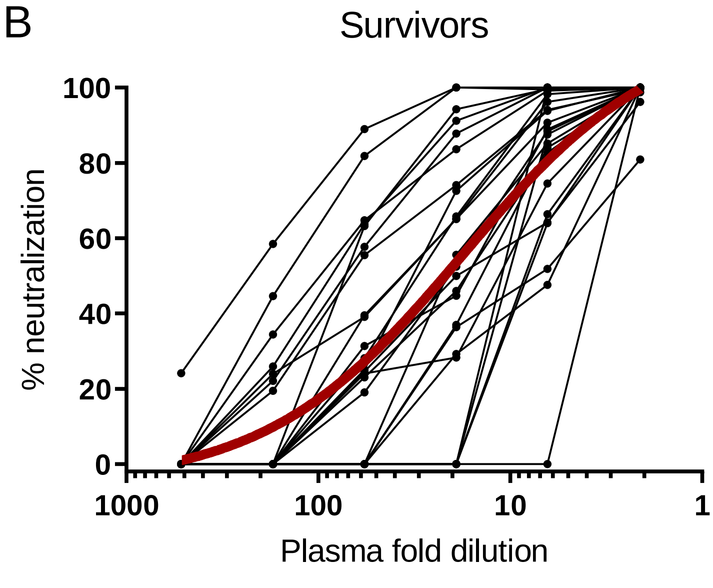

Figure S2: Individual neutralization curves. Shown are individual neutralization curves (thin black lines and dots) of the rVSV-ZEBOV vaccinees (**A**) and EVD-survivors (**B**). Thick lines represent the nonlinear regression of the median.

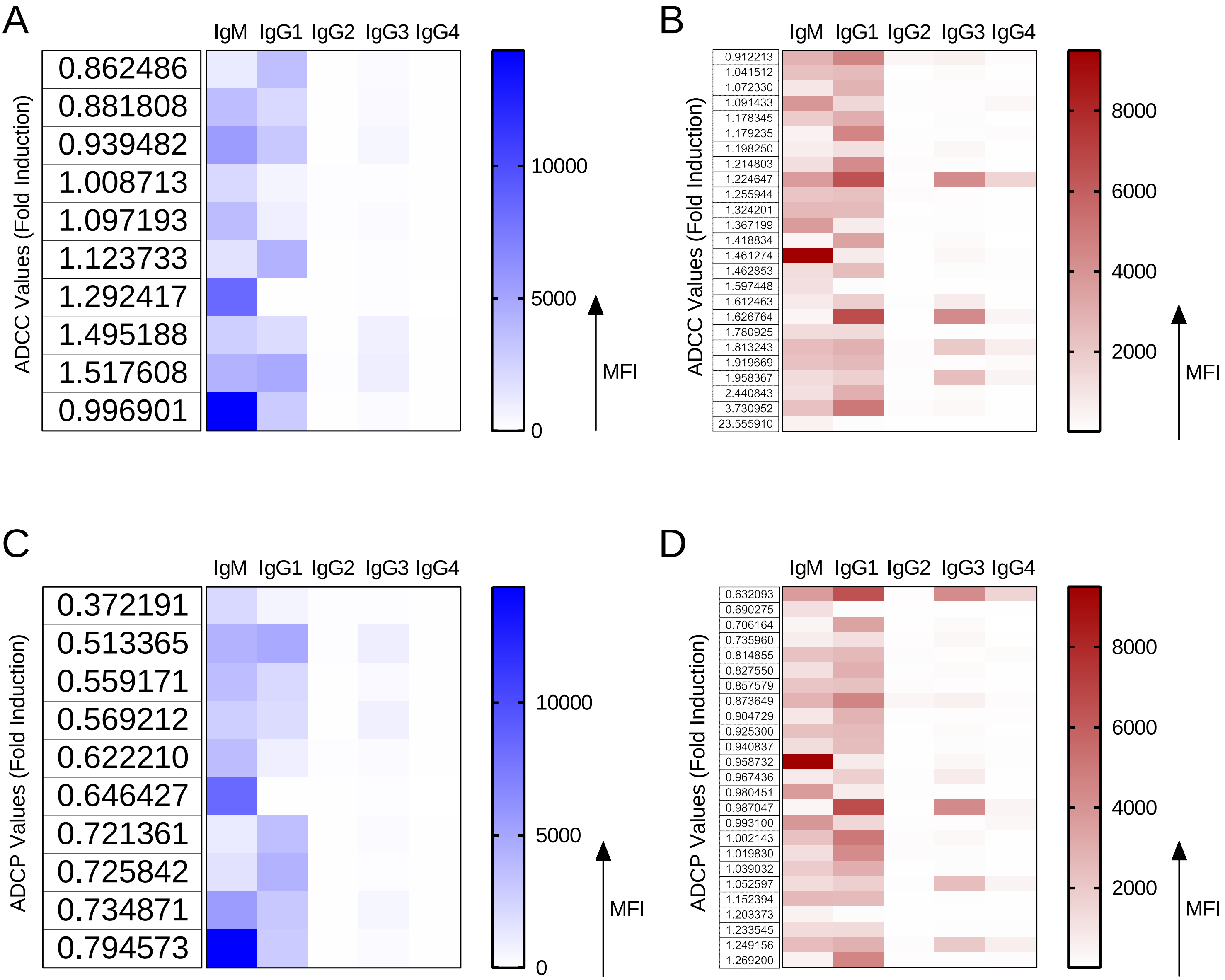

Figure S3: Correlation analysis. Spearman correlation analysis between ADCC (fold induction values) (A, B) and Ig subclass as well as ADCP (fold induction values) (C, D) and Ig subclass. Both analyses are displayed for vaccine recipients (A, C, blue) and EVD survivors (B, D, red).
